# Revision Cochlear Implantation via Middle Turn Insertion in Post-Traumatic Basal Turn Ossification: Revision cochlear implantation

**DOI:** 10.1007/s00405-026-10501-8

**Published:** 2026-08-31

**Authors:** Daniele Fasanella, Maurizio Guida, Maurizio Falcioni

**Affiliations:** 1 Department of Otolaryngology and Otoneurosurgery, Parma University Hospital, University of Parma, Parma, Italy; 2 Department of Otoneurosurgery and Lateral Skull Base microsurgery, Parma University Hospital, Parma, Italy

**Keywords:** Cochlear Implant, Revision Surgery, Subtotal Petrosectomy, Middle Turn Cochleostomy, Cochlear Ossification

## Abstract

**Background:**

Complete ossification of the basal turn of the cochlea represents a major challenge inrevision cochlear implantation, particularly in post-traumatic cases. Otic capsule violating temporal bone fractures need to be carefully managed to avoid future possible meningeal infections.

**Objective:**

To describe our surgical technique of middle turn cochleostomy combined with subtotal petrosectomy for revision cochlear implantation in the setting of total basal turn ossification due to an otic capsule-violating temporal bone fracture.

**Methods:**

We report a case of failed primary cochlear implantation due to post-traumatic ossification of the basal turn. Revision surgery was performed using subtotal petrosectomy to achieve wide exposure of the promontory. After removal of the previous device and confirmation of complete ossification of both scala tympani and scala vestibuli at the basal turn, promontory drilling was extended superiorly to identify and open the middle turn of the cochlea, allowing full electrode insertion.

**Results:**

Complete insertion of the new electrode array was achieved through the middle turn. Postoperative imaging confirmed appropriate positioning, facial nerve function was preserved, and the patient experienced improvement in auditory thresholds.

**Conclusions:**

In cases of total basal turn ossification, middle turn cochleostomy following subtotal petrosectomy represents a feasible and safe alternative to enable cochlear implantation in selected revision cases.

**Supplementary Information:**

The online version contains supplementary material available at https://doi.org/10.1007/s00405-026-10501-8.

## Key Message

1. Complete basal turn ossification does not necessarily preclude cochlear implantation when alternative cochlear access can be achieved.
2. In complex revision settings, subtotal petrosectomy may facilitate broad exposure of the promontory and surrounding structures.
3. Middle turn cochleostomy may allow successful electrode insertion when both scala tympani and scala vestibuli are completely ossified.
4. Careful drilling close to the modiolus is strongly suggested to avoid fractures and potential intraoperative CSF leakage.
5. Revision surgery after otic capsule violating fractures requires careful adaptation to altered anatomy and previous surgical modifications.

**Supplementary Information:**

The online version contains supplementary material available at https://doi.org/10.1007/s00405-026-10501-8.

## Introductory framing

We report a complex case of revision cochlear implantation in the setting of post-traumatic complete basal turn ossification, managed through subtotal petrosectomy and middle turn cochleostomy, supported by a surgical video supplement. The purpose of this “How I do it” report is to provide a practical step-by-step illustration of the surgical management and intraoperative decision-making process in a highly challenging revision setting.

### Case Presentation

We report a case of revision cochlear implantation using subtotal petrosectomy and middle turn cochleostomy, supported by a video supplement. Written informed consent was obtained from the participant included in the report and institutional board approval was obtained on August 4th, 2025 (protocol n. 34460, approved by Health Management board of Parma University Hospital, Parma, Italy).

**Fig. 1:**
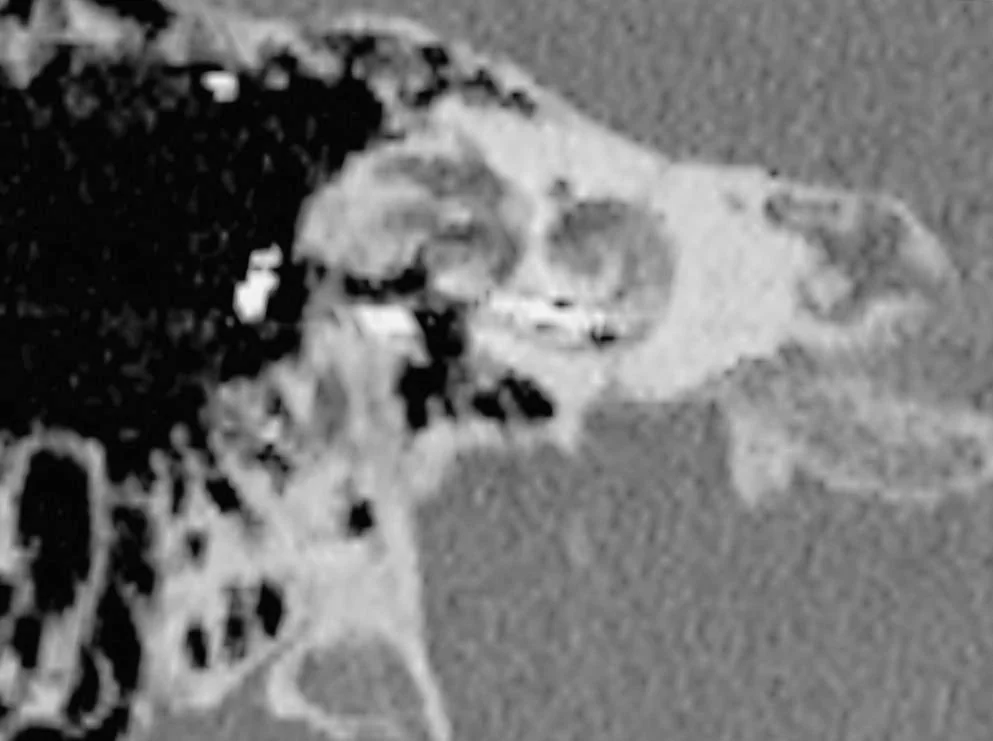
Postoperative temporal bone CT scan obtained after the index surgery performed at another institution, demonstrating incomplete insertion of the right cochlear implant array

A 47-year-old male sustained bilateral temporal bone fractures following cranial trauma, leading to bilateral anacusis. Facial nerve function was grade I according to the House-Brackmann scale. The patient had previously undergone bilateral cochlear implantation at another institution. The left-sided implant resulted in satisfactory outcomes, whereas the right-sided implant provided no auditory benefit. A postoperative high-resolution CT scan demonstrated correct electrode placement on the left side and incomplete insertion on the right side due to extensive ossification of the basal turn, as shown in Fig. 1.

The patient was subsequently referred to our center for evaluation and management of the non-functioning right cochlear implant.

In this revision setting, subtotal petrosectomy was selected to facilitate wide exposure of the promontory and surgical access during extensive drill-out. In addition, exclusion of the fractured otic capsule from the external environment may reduce the lifelong risk of meningitis associated with otic capsule violating fractures [1].

This technique consists of drilling all mastoid air cells, removing the ossicles (except for the stapes/footplate) and tympanic membrane, and obliterating the Eustachian tube with autologous material and the cavity with abdominal fat.

When subtotal petrosectomy is performed in the setting of extensive cochlear drill-out, several anatomical landmarks become particularly relevant and should be carefully identified:

- The tympanic and mastoid segments of the facial nerve, allowing safe drilling in close proximity to the nerve when required;
- The round and oval windows, useful as landmarks for identifying cochlear turns;
- The cochleariform process, which may help localize the apical turn;
- The carotid artery, whose identification can become important when drilling must be extended medially along the basal turn.

A Cochlear™ Nucleus^®^ CI612 electrode array (Cochlear Ltd., Sydney, Australia) was selected to facilitate insertion through the alternative cochlear access route.

### Surgical Technique

The surgery starts with a standard retroauricular incision and a 360° section of the external auditory canal. The canal is then closed as a blind sac by two-layer suture. The receiver-stimulator is identified and removed after sectioning the connecting cable.

The skin remnants of the external auditory canal are dissected medially toward the tympanic membrane. The posterosuperior canal wall, weakened by the fracture line, is subsequently drilled away, followed by a wide canaloplasty of the antero-inferior walls.

**Fig. 2:**
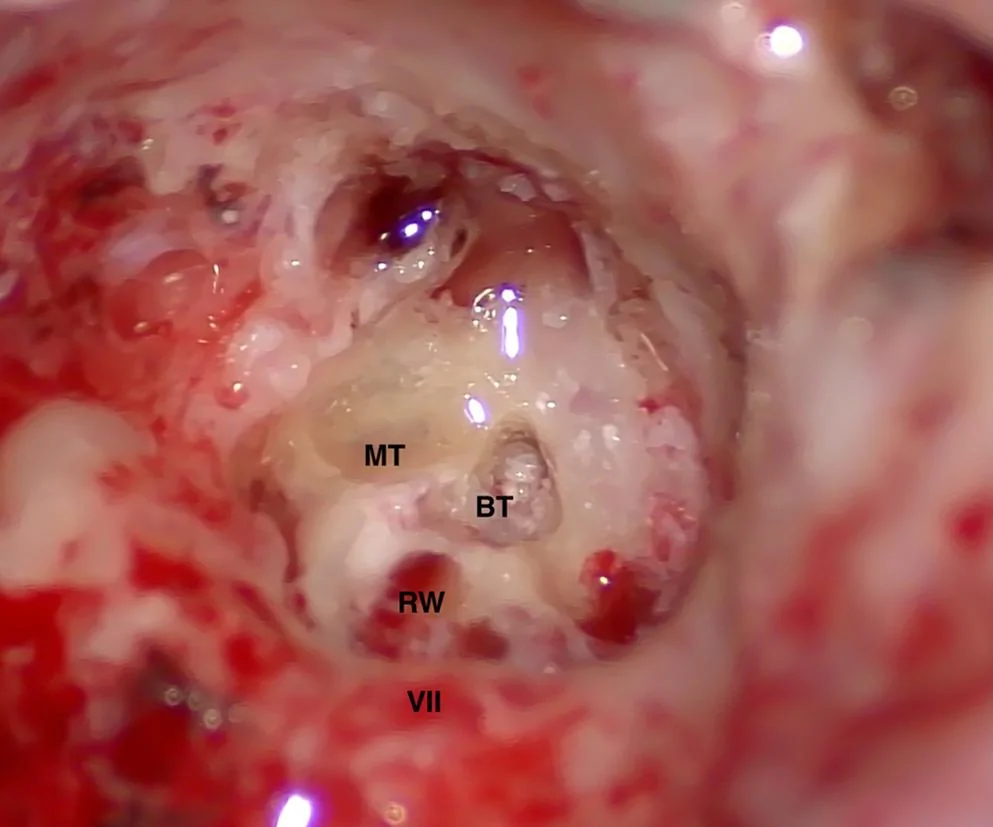
Intraoperative view showing complete ossification of both scala tympani and scala vestibuli of the basal turn. The middle turn of the cochlea can be seen in transparency. MT: middle turn, BT: basal turn, RW: round window, VII: Facial nerve

The incus is disarticulated from the stapes and removed. The tympanic membrane may then be removed en bloc together with the malleus and the medial skin of the external auditory canal. The facial ridge is lowered until optimal control of the round window region is obtained. When feasible, the previously inserted array may be left in place until the advanced stages of surgery, both as a landmark for the previous cochleostomy and to reduce the entrance of blood and bone dust into the cochlear lumen. In the present case, only five electrodes had originally been introduced into the cochlea. After visualization of the carotid artery, a first attempt was made to enlarge the cochleostomy anteriorly; however, the basal turn proved to be entirely ossified, and insertion of a dummy array was unsuccessful. An additional attempt to access the scala vestibuli through an anterosuperior drilling trajectory near the oval window also failed because of complete ossification, as shown in Fig. 2.

**Fig. 3:**
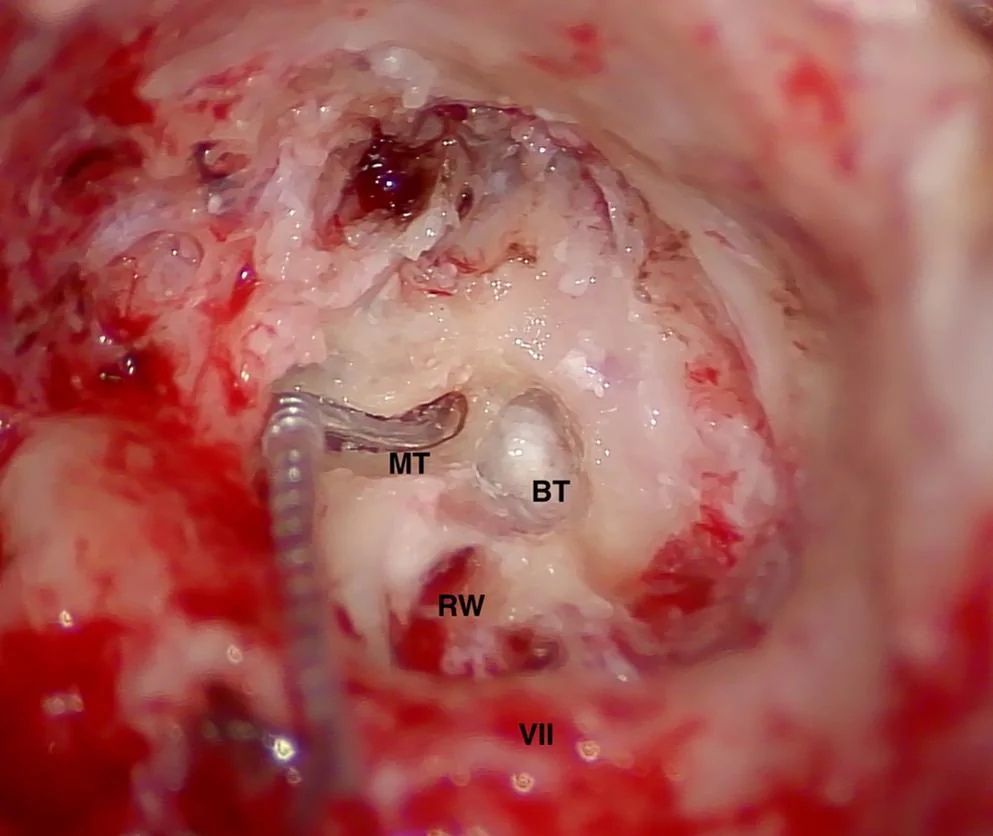
Middle turn cochleostomy allowing complete insertion of the electrode array after failure of basal turn access. MT: middle turn, BT: Basal turn of the cochlea, RW: round window, MT: middle turn, VII: Facial nerve

Promontorial drilling was therefore progressively extended superiorly until the middle turn of the cochlea became identifiable. Cochlear lumen access was achieved at this level, allowing complete insertion of the dummy electrode, as shown in Fig. 3.

**Fig. 4:**
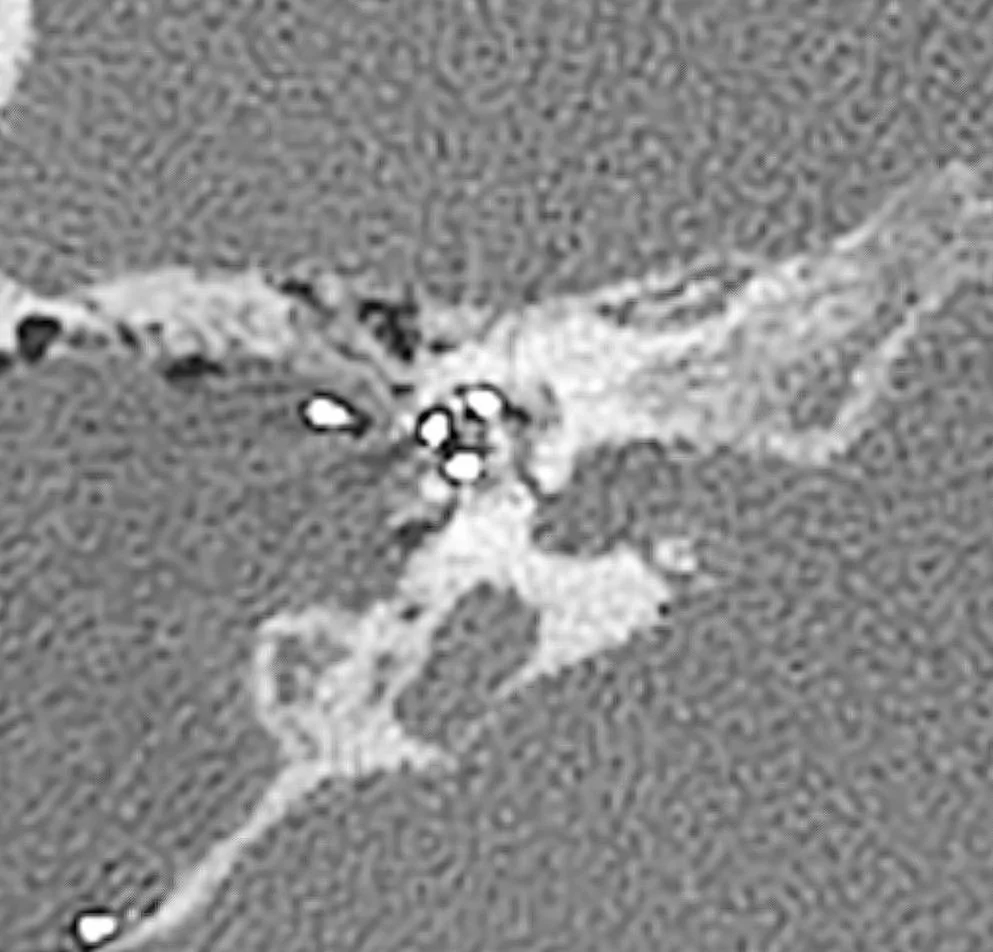
Postoperative temporal bone CT scan demonstrating complete insertion of the electrode array through the middle and apical cochlear turns with subtotal petrosectomy cavity obliteration using abdominal fat

The definitive electrode array (Cochlear™ Nucleus^®^ CI612, Cochlear Ltd., Sydney, Australia) was then inserted through the middle turn cochleostomy. Following intraoperative electrophysiological testing, the cavity was obliterated with abdominal fat. Figure 4 shows the postoperative temporal bone CT scan obtained one day after surgery. Embedded Video 1 and Embedded Video 2 show the entire surgical process.

### Subtotal Petrosectomy in Cochlear Implant surgery– Indications

Subtotal petrosectomy may represent a useful option in selected non-standard cochlear implantation cases, including anatomical variations, chronic ear disease, cholesteatoma, and severe cochlear ossification such as post-traumatic, post-meningitic, or otosclerotic cases [2].

In the setting of extensive cochlear ossification, subtotal petrosectomy may facilitate wider exposure of the promontory and surrounding structures, thereby allowing more extensive drill-out procedures when required [3]. In the present revision case, characterized by complete ossification of both scala tympani and scala vestibuli of the basal turn after otic capsule violating fracture, we considered subtotal petrosectomy the most appropriate strategy to achieve sufficient exposure while maintaining surgical control. Different approaches have been described in the literature, including extended basal turn drill-out, scala vestibuli insertion [4], middle turn cochleostomy, array insertion through middle fossa approach [5], circumodiolar drill-out techniques, and split-array strategies in cases of severe obliteration [6, 7]. Middle turn cochleostomy has previously been reported as a possible alternative when conventional basal turn access cannot be achieved because of complete ossification [8].

### How to avoid complications

- Meticulous removal of skin remnants is recommended to reduce the risk of iatrogenic cholesteatoma formation. En bloc removal, when feasible, may help minimize residual epithelial tissue, particularly near the bony annulus [9];
- Identification of the carotid artery may become particularly important during extensive promontorial drill-out;
- Keeping the previous array in place until the final stages of surgery may help preserve orientation toward the original cochleostomy and reduce blood and bone dust contamination of the cochlear lumen;
- Drilling close to the modiolus carries a risk of fracture and possible intraoperative CSF leak.

### Limitations

- Implant-related artifacts significantly limit the usefulness of DWI sequences for the follow-up. CT-scan is routinely used with less sensitive MRI sequences reserved for special cases.
- Subtotal petrosectomy is not routinely used for middle ear diseases, so and therefore may not be equally familiar to all otologic surgeons.
- Cochlear implantation in an extensive cochlear drill out setting for major basal turn ossification may fail to lead to satisfactory auditory outcomes, as well described in the current scientific literature [6].

### Specific perioperative considerations

- Post operative management of subtotal petrosectomy, with or without cochlear implantation, consists in performing a compressive head dressing medication for at least 2–3 days to avoid blood o serum collection in the cavity. Bleeding from the abdominal fat harvesting site may occur and should be carefully monitored.
- Resorbable sutures of the blind-sac closure must be kept in place; they are resorbed spontaneously after 2 months.

## Discussion

This report describes the surgical management of a complex cochlear implantation in the setting of complete post-traumatic basal turn ossification.

Cochlear ossification following temporal bone fracture represents a rare but challenging condition for cochlear implantation surgery. Extensive ossification involving both scala tympani and scala vestibuli may prevent standard electrode insertion through the basal turn and require alternative surgical strategies. Different approaches have been described in the literature, including extended basal turn drill-out, scala vestibuli insertion [4], middle turn cochleostomy, array insertion through middle fossa approach [5], circumodiolar drill-out techniques, and split-array strategies in cases of severe obliteration [6, 7]. Middle turn cochleostomy has previously been reported as a possible alternative when conventional basal turn access cannot be achieved because of complete ossification [8]. Similar reports have shown that access to the middle or apical cochlear turns may still permit electrode insertion and partial auditory rehabilitation even in extensively ossified cochleae. In this context, the present video contribution aims to provide a practical surgical illustration of the anatomical exposure and intraoperative steps required in such difficult situations. Subtotal petrosectomy was selected in the present case to facilitate broad exposure of the promontory and surrounding anatomical structures during extensive drill-out. Although alternative surgical approaches have been described, subtotal petrosectomy may provide substantial advantages in selected complex cases requiring aggressive promontorial drilling, particularly in the presence of distorted anatomy following temporal bone fractures [10]. In addition, exclusion of the fractured otic capsule from the external environment may contribute to reducing the long-term risk of meningitis associated with otic capsule violating fractures. In the present case, standard basal turn insertion as well as scala vestibuli access proved impossible because of complete ossification. Progressive superior promontorial drill-out ultimately allowed identification of the middle turn and successful electrode insertion through middle turn cochleostomy. A slim straight electrode array (CI612, Cochlear Ltd.) was selected in the present case to facilitate insertion through the alternative cochlear access route. The present case therefore illustrates how alternative cochlear access routes may still permit implantation despite complete basal turn obliteration. Functional outcomes following implantation in ossified cochleae remain variable in the literature [11]. Although pure-tone thresholds may improve substantially, speech discrimination outcomes are often less predictable, particularly in patients requiring non-standard insertion pathways or extensive drill-out procedures [10]. In our patient, postoperative pure-tone thresholds improved, whereas speech discrimination remained limited, representing a mixed but clinically meaningful functional outcome. Overall, this report highlights the complexity of cochlear implantation in extensively ossified cochleae and emphasizes the importance of adapting surgical strategy according to intraoperative anatomy and the extent of cochlear obliteration.

### Results

Postoperative imaging demonstrated satisfactory complete electrode positioning. Total surgical time was approximately 2.5 h.

Postoperative facial nerve function remained normal, and no facial nerve stimulation was observed after surgery. At one-year follow-up, the patient reported subjective auditory improvement. Audiological evaluation demonstrated satisfactory pure-tone thresholds, whereas speech discrimination performance remained limited, representing a mixed but clinically meaningful functional outcome.

### Conclusions

In cases of complete basal turn ossification, cochlear implantation may still be feasible through alternative insertion strategies such as middle turn cochleostomy. In selected complex revision settings requiring extensive promontorial drill-out, subtotal petrosectomy may provide advantageous surgical exposure and facilitate management of altered anatomy. In terms of audiological results, speech perception performance remains predominantly dependent on the contralateral implant, whereas the implant inserted through middle turn cochleostomy mainly contributes to environmental sound detection and sound localization.

This report aims to provide a practical surgical illustration of such a challenging scenario.

## Supplementary Information

Supplementary Material 1

Supplementary Material 2
